# Genomic Diversity, Antimicrobial Susceptibility, and Biofilm Formation of Clinical *Acinetobacter baumannii* Isolates from Horses

**DOI:** 10.3390/microorganisms11030556

**Published:** 2023-02-22

**Authors:** Johanna Rühl-Teichner, Lisa Jacobmeyer, Ursula Leidner, Torsten Semmler, Christa Ewers

**Affiliations:** 1 Institute of Hygiene and Infectious Diseases of Animals, Department of Veterinary Medicine, Justus Liebig University Giessen, 35392 Giessen, Germany; 2 Genome Sequencing and Epidemiology, Robert Koch Institute, 13353 Berlin, Germany

**Keywords:** multidrug resistance, international clones, biofilm-associated genes

## Abstract

*Acinetobacter (A.) baumannii* is an opportunistic pathogen that causes severe infections in humans and animals, including horses. The occurrence of dominant international clones (ICs), frequent multidrug resistance, and the capability to form biofilms are considered major factors in the successful spread of *A. baumannii* in human and veterinary clinical environments. Since little is known about *A. baumannii* isolates from horses, we studied 78 equine *A. baumannii* isolates obtained from clinical samples between 2008 and 2020 for their antimicrobial resistance (AMR), clonal distribution, biofilm-associated genes (BAGs), and biofilm-forming capability. Based on whole-genome sequence analyses, ICs, multilocus (ML) and core-genome ML sequence types (STs), and AMR genes were determined. Antimicrobial susceptibility testing was performed by microbroth dilution. A crystal violet assay was used for biofilm quantification. Almost 37.2% of the isolates were assigned to IC1 (10.3%), IC2 (20.5%), and IC3 (6.4%). Overall, the isolates revealed high genomic diversity. We identified 51 different STs, including 22 novel STs (ST1723–ST1744), and 34 variants of the intrinsic oxacillinase (OXA), including 8 novel variants (OXA-970 to OXA-977). All isolates were resistant to ampicillin, amoxicillin/clavulanic acid, cephalexin, cefpodoxime, and nitrofurantoin. IC1-IC3 isolates were also resistant to gentamicin, enrofloxacin, marbofloxacin, tetracycline, and trimethoprim/sulfamethoxazole. All isolates were susceptible to imipenem. Thirty-one multidrug-resistant (MDR) isolates mainly accumulated in the IC1-IC3 groups. In general, these isolates showed less biofilm formation (IC1 = 25.0%, IC2 = 18.4%, IC3 = 15.0%) than the group of non-IC1-IC3 isolates (58.4%). Isolates belonging to the same ICs/STs revealed identical BAG patterns. BAG *blp1* was absent in all isolates, whereas *bfmR* and *pgaA* were present in all isolates. At the level of the IC groups, the AMR status was negatively correlated with the isolates’ ability to form a biofilm. A considerable portion of equine *A. baumannii* isolates revealed ICs/STs that are globally present in humans. Both an MDR phenotype and the capability to form biofilms might lead to therapeutic failures in equine medicine, particularly due to the limited availability of licensed drugs.

## 1. Introduction

The Gram-negative bacterium *Acinetobacter* (*A*.) *baumannii* is a non-motile, non-fermentative, and non-fastidious bacterium that belongs to the family of *Moraxellaceae* [1]. It causes hospital- and community-associated infections, including urinary and respiratory tract infections, bacteremia, sepsis, and wound infections [2,3]. *A. baumannii* possesses several antimicrobial resistance (AMR) mechanisms that often provide the bacterium with a multidrug-resistant (MDR) phenotype. Among these are drug-modifying enzymes, efflux pumps, defects in membrane permeability, and the alteration of target sites [4]. Due to its MDR phenotype and its successful dissemination in different environments, *A. baumannii* has become one of six bacterial pathogens belonging to the ESKAPE group (*Enterococcus faecium*, *Staphylococcus aureus*, *Klebsiella pneumoniae*, *A. baumannii*, *Pseudomonas aeruginosa*, and *Enterobacter* spp.). Members of this group are frequently associated with treatment failures due to intrinsic and acquired antibiotic resistance [5].

In recent years, *A. baumannii* has also been recognized as a serious pathogen in animals, particularly due to its nosocomial dissemination in veterinary hospitals [6]. The isolates detected in animals often resemble clones that are circulating among humans. In the case of *A. baumannii*, nine international clones (ICs) have been identified to date, each representing at least one specific sequence type (ST): IC1 (ST1), IC2 (ST2), IC3 (ST3), IC4 (ST15), IC5 (ST79), IC6 (ST78), IC7 (ST25), IC8 (ST10), and IC9 (ST85). IC1, IC2, and IC3 are of particular interest because these account for the majority of nosocomial and community-acquired *A. baumannii* infections worldwide [7].

In 2011, Endimiani et al. [8] examined *A. baumannii* isolates from dogs, cats, and horses and discovered that the vast majority belonged to human-related international clones IC1 and IC2, corresponding to ST1 and ST2. Additionally, the presence of IC2/ST2 was detected in an equine *A. baumannii* isolate from 2002 by Zordan et al. (2011) [9]. These data provided the first indications of a possible transfer of *A. baumannii* isolates between humans and horses [9].

The ability of *A. baumannii* to form biofilms could be responsible for or promote urinary and respiratory tract infections in both human and veterinary medicine [10]. The biofilm allows *A. baumannii* to grow on abiotic surfaces such as stainless steel, polystyrene, and polycarbonate used for medical supplies, e.g., urinary catheters or endotracheal tubes [11,12,13]. The biofilm consists of a polymeric matrix that protects the bacteria from external factors. As a result, they appear to be more resistant to antimicrobial agents such as antibiotics or detergents [14,15]. Currently, there is little knowledge about the correlation between the biofilm-forming capability and AMR phenotype of *A. baumannii* isolates.

Various genes have been identified in *A. baumannii* that contribute to the virulence of the pathogen. Some of these virulence genes have been linked to the ability of *A. baumannii* to form biofilms, so-called biofilm-associated genes (BAGs). They encode for the chaperon-usher pilus Csu (*csuA/B/C/D/E*); the outer membrane protein A (*ompA*); the biofilm-associated protein (*bap*) as well as the Bap-like proteins (*blp1*, *blp2*); the quorum-sensing system AbaIR, consisting of the acyl-homoserine-lactone synthase AbaI (*abaI*) and the DNA-binding HTH-domain-containing protein AbaR (*abaR*); the polysaccharide poly-β-(1,6)-N-acetyl glucosamine (PNAG) encoded by gene cluster *pgaABCD*; and the two-component signal transduction BfmRS, consisting of response regulator BfmR (*bfmR*) and sensor kinase BfmS (*bfmS*) [13,16,17,18,19,20].

As there is scarce knowledge about the phylogeny and biology of *A. baumannii* isolates from horses, we investigated equine clinical *A. baumannii* isolates for their antimicrobial resistance and corresponding AMR genes and the biofilm formation ability and presence of BAGs. Moreover, international clones, STs, and core-genome multilocus sequence types (cgMLST) were determined to explore the strain’s diversity and possible overlaps with human isolates. Finally, the relationship between biofilm formation, clonal assignment, and AMR was investigated.

## 2. Materials and Methods

### 2.1. Bacterial Isolates

*A. baumannii* isolates (n = 78) from horses were randomly collected in our routine veterinary microbiological diagnostic laboratory from 2008 to 2020. The isolates were obtained from the genital and urinary tract (n = 21), wounds and abscesses (n = 19), the respiratory tract (n = 14), and various other clinical sites, including eyes, feces, gastrointestinal tract, organs, skin, hair, and hoof (n = 24). Species identification was performed with matrix-assisted laser desorption time-of-flight mass spectrometry (MALDI-TOF-MS) using the Microflex LT/SH mass spectrometer and the biotyper database (MALDI Biotyper V3.3.1.0) (both Bruker Daltonics, Bremen, Germany). Furthermore, the identification was confirmed by rMLST using the online tool “Identify species” provided by PubMLST (https://pubmlst.org/species-id (accessed on 30 December 2022)). All isolates were stored at −70 °C in Brain Heart Infusion Broth (Oxoid, Wesel, Germany) containing 30% glycerol.

### 2.2. Whole-Genome Sequencing

For whole-genome sequencing, DNA was extracted with the “Master Pure™ DNA Purification Kit” (Biozym Scientific GmbH, Hessisch Oldendorf, Germany). Sequencing libraries were prepared using the Nextera XT Library Preparation Kit (Illumina GmbH, Munich, Germany) for a 250 bp paired-end sequencing run on an Illumina MiSeq sequencer (Illumina Inc., San Diego, CA, USA) with a minimum coverage of 100-fold. FASTQ files were quality-trimmed before they were assembled de novo and annotated using SPAdes v.3.15.1 (http://cab.spbu.ru/software/spades/ (accessed on 30 December 2022)) and RAST v.2.0 (http://rast.nmpdr.org/ (accessed on 30 December 2022)).

### 2.3. Assignment of MLST Analysis and International Clones

Whole-genome sequences (WGS) were used to identify multilocus sequence types (MLST) by using the online tool MLST 1.6 (Pasteur scheme) provided by the Center for Genomic Epidemiology (CGE) [21,22]. International clones IC1, IC2, and IC3 were identified via multiplex PCR according to Turton et al. [23]. Moreover, a whole-genome-based comparison of all *A. baumannii* isolates with representative genomes of IC1 to IC9 was performed to categorize the studied isolates into the different clonal groups. A neighbor-joining tree was created using Ridom SeqSphere+ v. 7.7.0. In the case that isolates could not be assigned to any IC, their genomes were submitted to BacWGSTdb 2.0, a whole-genome bacterial sequence typing and source tracking repository [24]. Using the tool “single genome analysis”, closely spaced isolates were identified from the database based on an SNP strategy with a threshold of 1000 SNPs. The first two genomes that matched with our isolates were included in an SNP-based genome comparison.

### 2.4. Antimicrobial Susceptibility Testing

Minimal inhibitory concentration (MIC) testing was carried out using the VITEK^®^ 2 system (AST card GN-97; bioMérieux, Nürtingen, Germany). Antibiotics tested were ampicillin (AMP), amoxicillin–clavulanic acid (AMC), cephalexin (CEX), ceftiofur (CTU), cefpodoxime (CPD), imipenem (IPM), amikacin (AMK), gentamicin (GEN), enrofloxacin (ENR), marbofloxacin (MAR), tetracycline (TET), nitrofurantoin (NIT), chloramphenicol (CHL), and trimethoprim/sulfamethoxazole (SXT). In the absence of veterinary-specific clinical breakpoints for *Acinetobacter* spp. isolates, human-specific clinical breakpoints from CLSI document M100 [25] were applied whenever possible. For AMC, CPD, NIT, and CHL, breakpoints for *Enterobacterales* [25] were used; for ENR and MAR we used breakpoints provided for ciprofloxacin [25]. For AMP and CEX, breakpoints for *Enterobacterales* as listed by EUCAST [26] were applied. Intrinsic resistance was assumed according to the definitions described in the CLSI guidelines [25]. For AST performed with the VITEK^®^ 2 system, *Escherichia coli* ATCC^®^ 25922 and *Pseudomonas aeruginosa* ATCC^®^ 27853 served as quality control strains.

### 2.5. Resistance Genes and Biofilm-Associated Genes

WGS data were used to identify AMR genes by using the online tools ResFinder 4.1, provided by the CGE, and “multiple genome analysis” provided by BacWGSTdb 2.0 [24,27,28,29]. Intrinsic oxacillinase (OXA) genes were compared by using the Geneious Version 8.1.9 software (Biomatters Ltd., Auckland, New Zealand) based on all OXA amino acid sequences for *A. baumannii* available in NCBI [30]. Protein sequences of the OXA variants that were not 100% identical to known OXA variants were submitted to NCBI, and new allele numbers were assigned. Screening for BAGs was performed by using the online tool “multiple genome analysis” provided by BacWGSTdb 2.0 [24,31]. Prediction of AMR genes and BAGs was established with a threshold of 90% each for nucleotide identity and length coverage.

### 2.6. Biofilm Assay

Biofilm production was quantified by a crystal violet assay slightly modified from O’Toole et al. (2011) [32]. Overnight cultures of *A. baumannii* isolates were harvested from blood agar plates (blood agar base by Merck Chemicals, GmbH, Darmstadt, Germany, supplemented with 5% sheep blood) and incubated for at least 18 h at 37 °C in 3 mL Luria–Bertani (LB) broth and 3 mL M63 minimal medium on a shaker. Afterwards, the bacteria were harvested and the OD_600_ was adjusted to 0.05. Biofilms were cultured in 96-well microtiter plates (F-Profile, Rotilabo^®^ by Carl Roth, Karlsruhe, Deutschland) sealed with Breathe-Easy^®^ sealing membrane (Diversified Biotech) for 24 h and 48 h at 37 °C. After incubation, supernatants were removed, and each well was washed twice using distilled water. The bacterial biofilms were stained using 0.1% (*w*/*v*) crystal violet (Merck, Darmstadt, Deutschland) in 0.9% (*w*/*v*) NaCl solution. After two washing steps, the bound crystal violet was eluted using 90% ethanol. The amount of eluted crystal violet was used as a surrogate for the produced mass of biofilm, and the concentration was determined photometrically by using the Multiskan FC (Thermo Fisher Scientific) at OD_570_ and OD_595_. *A. baumannii* strains ATCC 19606 and ATCC 17978 were used as positive and negative controls, respectively [33,34]. All assays were carried out in triplicate, and the results were averaged. For further statistical analysis, the degree of the specific biofilm formation (SBF) was determined, and the specific biofilm formation threshold (SBF_T_) was defined as three standard deviations above the mean SBF of the negative control (ATCC 17978). The results were classified as follows [35]:
Non-biofilm producerSBF ≤ SBF_T_;Weak biofilm producerSBF_T_ < SBF ≤ 2× SBF_T_;Moderate biofilm producer2× SBF_T_ < SBF ≤ 4× SBF_T_;Strong biofilm producer4× SBF_T_ < SBF.

### 2.7. Statistical Analysis

Statistical analyses were performed by using the SPSS Statistics version 27 software (IBM, Armonk, NY, USA). All statistical tests were two-sided, and *p* < 0.05 was considered as statistically significant. The chi-square test was used to analyze the differences in biofilm formation among groups. The two-sample *t*-test and two-way ANOVA were used to conduct multiple comparisons between the clinical origin and the clonal linage or sequence types of the isolates.

## 3. Results

### 3.1. Phylogenetic Analysis

More than one-third (37.2%) of the 78 *A. baumannii* isolates from horses could be assigned to the international clones IC1 (10.3%), IC2 (20.5%), and IC3 (6.4%). The remaining 49 isolates were regarded as “non-IC1-IC3”, as they could not be assigned to IC1, IC2, or IC3 and ST1, ST2, or ST3, respectively, based on the applied PCR assay and MLST analysis (Table 1).

Based on the comparison of the core genome of *A. baumannii* isolates and representative *A. baumannii* isolates of IC1-IC9, other ICs could also not be assigned to the equine *A. baumannii* isolates. The majority of non-IC1-IC9 isolates were singletons, i.e., no related genome could be found in the public database as analyzed with the online tool BacWGSTdb (http://bacdb.cn/BacWGSTdb/Tools.php (accessed on 30 December 2022)). A phylogenetic tree based on the comparison of 2.390 core-genome genes of the 78 *A. baumannii* isolates from horses and 18 representative *A. baumannii* genomes belonging to international clones IC1-IC9 is provided in Supplementary Figure S1. Following the Pasteur MLST scheme, we identified 51 different STs, indicating an overall high diversity among isolates from horses. In agreement with the IC assignment, the most abundant STs were ST2 (19.2%), ST1 (10.3%), and ST3 (6.4%). In addition, 22 new STs (ST1723–ST1744) were identified.

Notably, IC1/ST1 and IC2/ST2 were found to be more prevalent in wound infections (IC1/ST1 = 63%, IC2/ST2 = 38%), whereas non-IC1-IC3 isolates were mainly found in infections of the genital tract (37%) and the respiratory tract (22%). However, because in some cases the number of isolates per clinical origin is too low, we cannot provide a statistical support for this observation.

### 3.2. Antimicrobial Susceptibility and AMR Genes

All 78 equine *A. baumannii* isolates were resistant to AMP, AMC, CHL, CEX (all intrinsic according to CLSI [25]), and NIT. Resistance to CTU, CPD, GEN, and ENR/MAR was identified in 98.7%, 94.9%, 38.5%, and 37.2%/35.9% of the isolates, respectively. None of the isolates was resistant to the carbapenem antibiotic IPM. Thirty-one isolates (38.5%) were classified as MDR (i.e., resistant to substances of three or more antimicrobial classes) according to the definition set by Magiorakos et al. (2012), where intrinsic resistances of the respective species are not considered [36]. About two-thirds (66.7%) of the isolates were susceptible to TET, and 62.8% revealed susceptibility to SXT. Overall, IC1-IC3 isolates revealed higher resistance rates compared to non-IC1-IC3 isolates. Of the 29 IC1-IC3 isolates, all were resistant to GEN and ENR, 96.6% to MAR, 93.1% to SXT, and 82.8% to TET. In contrast, only 4.1% of the 49 non-IC1-IC3 isolates showed resistance to TET and SXT, and 1 isolate was GEN-resistant.

The most common AMR genes identified among the 78 equine isolates belonged to the group of β-lactams (*bla*_OXA-51-like_, 100%; *bla*_ADC-25_, 98.7%; and *bla*_TEM-1D_, 30.8%) followed by aminoglycosides (different genes accounting for 5.1% to 32.1%), sulfonamides (*sul1*, 35.9% and *sul2*, 2.6%), phenicols (*catA1*, 26.0%; *ABUW 0982*, 100%), and tetracyclines (*tet*(A), 10.3%; *tet*(B) and *tet*(39), 1.3%) (Table 2). Insertion sequences prior to *bla*_OXA-51-like_ genes were not identified. Almost in accordance with the phenotypic resistance data, non-IC1-IC3 isolates only rarely (2 of 49 isolates) possessed acquired AMR genes. The first isolate (IHIT18056), obtained from the skin region of the hoof in 2011, was positive for *tet*(B), *sul2*, and *aph(3′)-Ia* and showed phenotypic resistance to TET and SXT (Supplemental Table S1). The second isolate (IHIT32845), cultivated from a wound swab in 2016, possessed *tet*(39), *sul2, aph(3`)-Ia*, *aph(3`)-Ic*, *aph(6)-Id*, and *aac(3)-Ia*; was resistant to TET, SXT, and GEN; and was classified as MDR. Notably, among 31 MDR isolates, 29 (93.6%) belonged to IC1-IC3. Although all IC3 isolates were MDR, they lacked *bla*_TEM-ID_, *tet*(A), and *tet*(B) that were regularly present in IC1 (*bla*_TEM-ID_ and *tet*(A)) and IC2 isolates (*bla*_TEM-ID_ and *tet*(B)) (Supplementary Table S1).

### 3.3. Instrinsic Oxacillinase Variants

**Figure 1:**
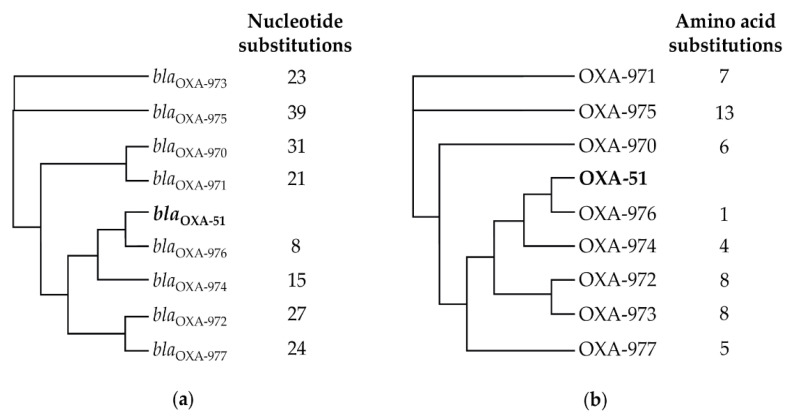
Neighbor-joining trees of the eight novel (**a**) *bla*_OXA-51_-like variants in comparison to *bla*_OXA-51_ and (**b**) OXA-51-like variants in comparison to OXA-51 indicating the number of nucleotide and amino acid substitutions, respectively. Alignment trees were performed by using “Geneious Tree Builder” provided by Geneious 8.1.9 (Biomatters, Auckland, New Zealand). NCBI Reference Sequences for *bla*_OXA-970_ to *bla*_OXA-977_: NG_077991.1 to NG_077998.1.

Overall, 34 different OXA variants were determined, all belonging to the OXA-51 family, which is characteristic for *A. baumannii* [37]. As expected, isolates belonging to IC1, IC2, and IC3 harbored oxacillinases OXA-69, OXA-66, and OXA-71, respectively. However, OXA-69 was also identified in the non-IC1-IC3 genital tract isolate IHIT30088 that was assigned to a novel ST (ST1737). Moreover, OXA-71 was determined in two non-IC1-IC3 isolates (IHIT27297 from the genital tract and IHIT31619 from the respiratory tract) that belonged to the novel ST1732 and to ST275. Interestingly, in a phylogenetic tree based on the comparison of 2390 core-genome genes of 78 *A. baumannii* isolates from horses and 18 representative *A. baumannii* genomes belonging to IC1-IC9, IHIT30088 clustered far away from IC1, while IHIT27297 and IHIT31619 likewise clustered outside the IC3 group (Supplementary Figure S1). Among the 49 non-IC1-IC3 isolates, the most frequently detected OXA variants were OXA-104 (n = 6), OXA-51 (n = 4), and OXA-65 (n = 4), while the remaining 30 variants were represented by either one or two isolates. Eight novel OXA-51-like variants were identified, namely, OXA-970 to OXA-977 (GenBank Accession nos.: QWA20169.1–QWA20176.1). These variants revealed 8 (*bla*_OXA-976_) to 39 (*bla*_OXA-975_) nucleotide substitutions (Figure 1a) and 1 (OXA-976) to 13 (OXA-975) amino acid substitutions (Figure 1b) compared with the *bla*_OXA-51_ (GenBank: AJ309734.2) and OXA-51 (GenBank: CAC83905.2) reference sequences. The similarity of the novel OXA variants to OXA-51 and *bla*_OXA-51_ was 99.9%.

### 3.4. Biofilm Formation and Biofilm-Associated Genes (BAGs)

Using the crystal violet assay, the SBF was determined for all 78 *A. baumannii* isolates. In terms of median and maximal SBF, we observed an overall stronger biofilm formation in M63 compared to LB medium (Table 3).

For further analysis, the SBF_T_ for each medium and incubation time was determined as described before. The following values were obtained: LB 24 h = 5.54; LB 48 h = 6.76; M63 24 h = 11.81; and M63 48 h = 13.98. For both media, a significant difference in the SBF_T_ at the two incubation times was not observed (LB 24 h and 48 h: *p* = 0.579; M63 24 h and 48 h: *p* = 0.087).

**Figure 2:**
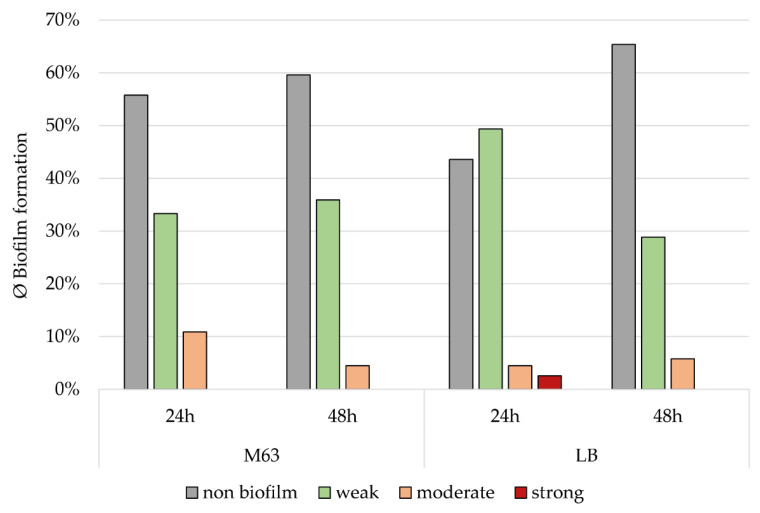
Occurrence of the individual classes (non-biofilm, weak, moderate, and strong) among 78 equine *A. baumannii* isolates in LB and M63 media after 24 or 48 h of incubation. Comparison of the two media at the same incubation time point revealed a significant difference in biofilm formation (for both, *p* < 0.001). However, within the same medium, there was no significant difference between the incubation times (LB: *p* = 0.579; M63: *p* = 0.087).

The classification of the isolates into the categories non-biofilm, weak, moderate, and strong biofilm producer was based on the SBF values and the corresponding SBF_T_ as described before (data are provided in Supplementary Table S1). Based on this classification, it was found that, despite the generally higher SBF values in M63, biofilm formation was strongest in LB after 24 h of incubation. In general, biofilm formation decreased over time, but this decrease was more evident in LB than in M63 after 48 h. In both media, the number of non-biofilm producers was higher after 48 h than after 24 h. While 56.4% of the strains were above the SBF_T_ in LB after 24 h, it was only 34.6% after 48 h. This results in a difference of 21.8% between the two incubation times. In M63, this difference was only 3.8% (24 h = 44.2%, 48 h = 40.4%) (Figure 2).

Of the 18 BAGs tested, only *blp1*, which encodes for the biofilm-associated-like protein 1, was not detected in any of the 78 equine *A. baumannii* isolates (Table 4 and Supplementary Table S1). Some BAGs were either regularly (*bfmR* and *pgaA*) or very frequently (*abaI*, *abaR*, *bfmS*, *csuA-E*, and *pgaB-D*), i.e., in >92% of the isolates, detected (Table 4). Only the genes *blp2* (75.6%), *bap* (55.1%), and *ompA* (55.1%) were identified in lower percentages. Isolates belonging to the same ICs or STs basically shared the same or highly similar BAG patterns (Supplementary Table S2). IC1 and IC3 isolates always lacked the genes *bap* and *ompA*, which were present in all IC2 isolates. The number of BAGs according to IC assignment decreased from IC2 (mean number of 16.9 BAGs) to IC1 (15.0), IC3 (14.4), and non-IC1-IC3 isolates (13.6).

### 3.5. Correlation of Biofilm Formation with ICs/STs, Antimicrobial Susceptibility, and Resistance Genes

**Figure 3:**
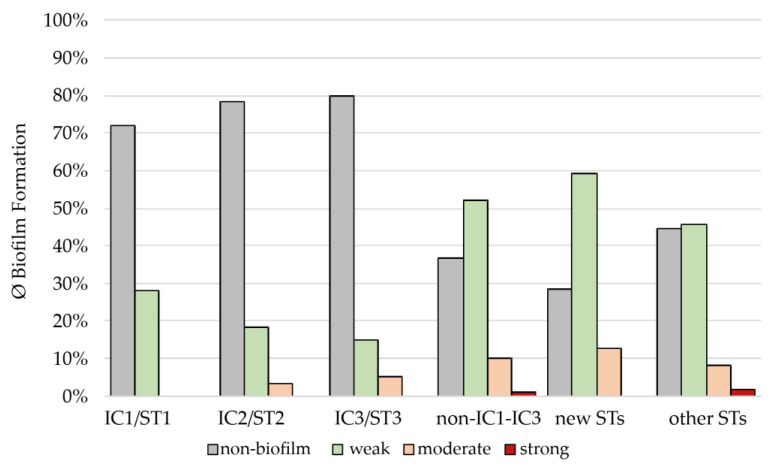
Occurrence of the individual classes (non-biofilm, weak, moderate, and strong biofilm) of the 78 equine *A. baumannii* isolates based on their determined STs or ICs. New STs = ST1723-ST1744 (n = 22 isolates); other STs = STs not belonging to ST1, ST2, ST3, or new STs (n = 28 isolates); all *p*-values > 0.1, except for other STs (*p* < 0.05).

Overall, *A. baumannii* isolates that belonged to IC1-IC3 showed a lower biofilm formation than non-IC1-IC3 isolates (*p* = 0.02). Only 28.1% of IC1, 18.6% of IC2, and 20.0% of IC3 isolates revealed a weak or moderate biofilm formation, while the remaining isolates were below the SBF_T_ and were thus defined as non-biofilm producers. This was in contrast to the 49 non-IC1-IC3 isolates, comprising non-ST1-ST3 isolates and those belonging to novel STs. Here, more than half of the isolates (63.3%) showed a biofilm-forming capability that was above the calculated SBF_T_. Of these, 52.1% were categorized as weak, 10.2% as moderate, and 1.0% as strong biofilm producers. More than half (59.1%) of the isolates assigned to novel STs showed weak biofilm formation, while 12.5% were moderate biofilm producers. Among 28 isolates assigned to other STs, 45.5% showed weak, 8.0% moderate, and 1.8% strong biofilm production (Figure 3).

**Figure 4:**
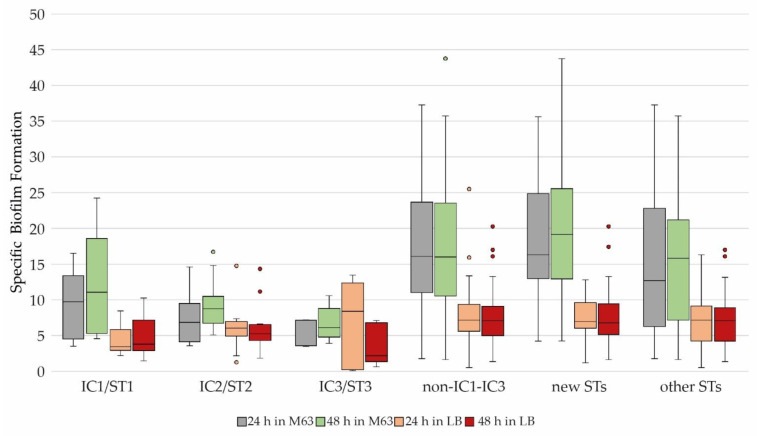
Distribution of the SBF of 78 equine *A. baumannii* isolates based on their STs/ICs depending on incubation time and medium used. Number of isolates: IC1/ST1 = 8, IC2/ST2 = 16, IC3/ST3 = 5, non-IC1-IC3 = 49, other STs = 29, new STs = 21; other STs = non-ST1, -ST2, -ST3; new STs = ST1723-ST1744. All ICs/STs: *p* < 0.05, except for IC3/ST3: *p* = 0.613.

Notably, all clonal lineages except for IC3 showed a higher SBF value in LB than in M63 (IC1/ST1: *p* = 0.013; IC2/ST2: *p* = 0.032; IC3/ST3: *p* = 0.613; new STs: *p* < 0.001; other STs: *p* < 0.001). In addition, the SBF values of IC1 to IC2 isolates scattered much more in LB versus M63 medium, while there was an overall higher scattering of SBF values among non-IC1-IC3 isolates (Figure 4).

We further explored a possible relationship between biofilm formation and phenotypic resistances of the isolates. For this purpose, acquired resistances to CTU, IPM, AMK, GEN, ENR, TET, and SXT were considered. Intrinsic resistances were excluded from the analysis, and of the fluoroquinolone antibiotics, only ENR was considered. Except for one isolate (susceptible to all seven antibiotics), the remaining isolates were resistant to a minimum of one (CTU) to a maximum of five (CTU, GEN, ENR, TET, and SXT) antibiotics. None of the isolates was resistant to IPM or AMK. Irrespective of the medium and incubation time used for the biofilm assays, non-biofilm producers revealed the highest number of resistances, followed by weak biofilm producers (Table 5). In detail, 41.4% to 46.3% of the non-biofilm and 7.7% to 23.3% of the weak biofilm producers were resistant to five antibiotics, namely, CTU, GEN, ENR, TET, and SXT. In contrast, moderate and strong biofilm producers showed varying resistances, with a maximum resistance to 5 (moderate) and 2 (strong) antibiotics.

## 4. Discussion

*A. baumannii* is a globally distributed pathogen associated with infectious diseases in humans and animals. The frequent resistance to a number of antibiotics belonging to different classes drastically limits the antimicrobial treatment options for *A. baumannii* infections. So far, *A. baumannii* isolates obtained from clinical diseases in horses have rarely been investigated *in-depth*, i.e., with respect to their phylogenetic diversity, antimicrobial susceptibility, AMR genes, and capability to form a biofilm. Few studies screened equine *A. baumannii* isolates, often in small to moderate numbers, for their phenotypic and genotypic resistances, and even fewer performed WGS-based analysis for a detailed molecular characterization of the isolates [4,8,9,38,39,40].

In the present study, more than one-third (37.2%) of the 78 equine *A. baumannii* isolates obtained from different clinical sites, predominantly from the genitourinary system (26.9%), wounds/abscesses (24.4%), and respiratory tract (17.9%), belonged to the globally distributed international clones IC1 to IC3, while the remaining isolates were non-IC1-IC9. So far, IC1 and IC2 isolates have only sporadically been reported from clinical samples of horses, e.g., in Switzerland (3 × IC1 and 1 × IC2) and Germany (1 × IC2) [8,9]. *Acinetobacter baumannii* were also cultivated from jugular catheter tips collected from horses hospitalized in a university clinic in Belgium [39] and in the nostril swabs, feces, and wounds of horses at a university clinic in Germany [38]. Neither study provided information about IC assignment. However, our findings clearly demonstrate that *A. baumannii* isolates belonging to ICs identified among human patients are also emerging in horses and, as recently shown, other animals [2,3,6,7,41].

About one-third (62.8%) of our isolates could not be assigned to a known IC, even after a comparison of their genomes with reference genomes of isolates previously grouped to international clones IC4 to IC9 (Supplementary Figure S2). In general, clonal lineages IC4 to IC6 and IC9 are rarely found in animals. Our group recently demonstrated one IC8 isolate from a dog (Italy) and nine IC7 (ST25) isolates from dogs and cats (France, Italy, and Germany), all producing carbapenemases [42]. After ST1 and ST2, ST25 represents one of the most relevant sequence types globally distributed in the human domain. Moreover, reports from France and Switzerland hint towards increasing rates of IC7/ST25 *A. baumannii* isolates in addition to IC1-IC3 isolates among companion animals [43,44]. According to our data, this lineage has not yet reached horses in Germany, although this should be further monitored.

Overall, the equine *A. baumannii* isolates from this study were phylogenetically diverse but exhibited clonal lineages that are globally distributed in the human clinical setting [6,7]. Our data suggested further parallels to the human domain as all IC1-IC3 isolates could be classified as MDR [36,45]. In 2000, Vaneechoutte et al. found similar results in seven *A. baumannii* isolates from 23 horses. All the isolates showed resistance to AMX, AMC, CTU, TET, and potentiated sulfonamides, while three isolates were resistant to GEN [38]. In a retrospective analysis, 23 of 24 equine *A. baumannii* isolates obtained from horses in an equine hospital between 2012 and 2015 could be classified as MDR based on their phenotypic resistances [46]. Moreover, Walther et al. (2018) reported that one of nine *A. baumannii* isolates from horses was resistant to CTU, GEN, TET, SXT, and tobramycin and thus revealed an MDR phenotype [40]. In our study, the finding of two MDR isolates outside of the IC1-IC9 group indicates that other lineages might emerge which are capable of acquiring AMR genes and developing an MDR phenotype.

The occurrence of AMR genes was almost indicative of the phenotypic resistances identified. Although the chloramphenicol acetyltransferase gene *catA1* was detected in only 26.9% of the isolates, we identified *ABUW_0982* in all isolates. This gene was recently described as a primary determinant of intrinsic CHL resistance in the human MDR *A. baumannii* strain 5075 [47]. The 30 GEN-resistant isolates harbored at least one aminoglycoside-modifying enzyme, and all 26 TET-resistant isolates carried either *tet*(A) or *tet*(B). Phenotypic resistance to MAR and ENR was based on mutations in GyrA (S83L) and ParC (S80L) [48], and the lack of IPM resistance was due to the absence of *ISAba1* upstream of the regularly found *bla*_OXA-51-like_ gene [49]. Previous studies likewise showed that in contrast to isolates from humans or small animals, *A. baumannii* isolates from horses are mostly susceptible to carbapenems [8,50,51]. Van Spijk et al. detected IPM resistance in 1 of 24 equine *A. baumannii* isolates [46]. In a study by Rafei et al. (2015), 597 samples were collected from the environment (soil and water), animals, and food products from 2012 to 2013. *A. baumannii* was found in 42 samples, of which one isolate (ST294) from a horse mouth swab was resistant to doripenem [52]. In 2012, Smet et al. detected two IPM-resistant, OXA-23-producing *Acinetobacter species* (later defined as *A. gandensis*) isolates from fecal samples of horses in Belgium [53].

As recently shown for human clinical *A. baumannii*, the biofilm formation capability of equine *A. baumannii* also tends to be negatively correlated with AMR and assignment to ICs [33]. Our data indicate that equine *A. baumannii* isolates that belonged to either IC1, IC2, or IC3 and represented MDR isolates were mostly not able to produce biofilm. Among these isolates, only a weak biofilm formation could occasionally be demonstrated in both LB and M63 media. Various studies previously reported that MDR *A. baumannii* isolates tended to form weaker biofilms than non-MDR isolates. For *A. baumannii* isolates from human patients in China, Qi et al. noted that 79.4% of the strong biofilm producers (n = 63) were non-MDR and 20.6% were MDR/XDR (extremely drug-resistant). In contrast, only 20.6% (n = 186) of weak and 8.7% (n = 23) of non-biofilm producers were non-MDR isolates [33]. In 2018, Rahimi et al. obtained similar results when investigating 80 *A. baumannii* isolates from patients in hospitals in Iran. Again, 90% (n = 27) of non-MDR isolates and 35% (n = 6)/21% (n = 7) of MDR/XDR isolates were strong biofilm producers [54]. Espinal et al. (2012) reported that non-biofilm-producing human *A. baumannii* isolates harbored significantly more AMR genes than biofilm producers [14].

In contrast, Yang et al. also noted an opposite effect and showed a stronger biofilm formation in MDR isolates than in non-MDR isolates. However, the correlation of biofilm formation and AMR was determined for each antibiotic individually which differs from the aforementioned studies [55]. Such controversial data clearly warrant further research in this field to gain a deeper understanding of the relationship between biofilm formation and AMR. A deeper understanding of the molecular mechanisms of biofilms, which may serve as a bacterial survival strategy, could provide novel insights into therapeutic and prevention measures to combat *A. baumannii* biofilm-related infections.

Due to their suggested impact on biofilm formation, we investigated the presence of BAGs in our isolates. Of the 18 BAGs tested, only *blp1* could not be detected in any isolate. In contrast, *pgaA* and *bfmR* were present in all isolates. Among IC1 and IC3 isolates, the genes *bap* and *ompA* were regularly absent, indicating a lineage-specific distribution of some BAGs. We could not find statistical support for a correlation between the presence of BAGs and the extent of biofilm formation. In various studies, the influence of individual BAGs on biofilm formation was investigated by deactivating or knocking out a single BAG from the chromosomes of *A. baumannii* isolates. Gregorio et al. could show that biofilm formation was lower after inactivation of *blp1* and *blp2* [56]. Moreover, Gaddy et al. demonstrated that inactivation of *ompA* prevented the biofilm formation of an *A. baumannii* isolate [57].

Comparing the biofilm formation with clonal linages, it turned out that not only did our IC1-IC3 isolates differ from the non-IC1-IC3 isolates in that they showed less biofilm formation in both media, but that the IC3 isolates were again more different from the other clonal lineages, as these isolates showed greater biofilm formation in M63 than in LB. The correlation between assignment to an IC and strength of biofilm formation has previously been studied with 80 human clinical *A. baumannii* isolates. Using a crystal violet staining method, *A. baumannii* belonging to IC1 and IC2 were found to form less biofilm than those not belonging to any IC [54].

## 5. Conclusions

In this study, we provide extensive phenotypic and genotypic typing of a considerable collection of *A. baumannii* isolates obtained from clinical samples of horses. The frequent identification of international clones and multilocus sequence types that are globally distributed in humans raises questions about the source of these isolates. Currently, it seems most likely that there is a spillover from humans and their environment to horses, as has already been suggested for companion animals. A multidrug-resistant phenotype might lead to therapeutic failures in equine medicine, particularly due to the limited availability of licensed drugs. The fact that less resistant equine isolates might escape from efficient antibiotic treatment by forming biofilms is another worrying finding. Future research in this field should further explore the complex interplay between phylogenetic background, antimicrobial resistance, and biofilm production in *A. baumannii* isolates from horses as well as from other animals and humans.

## Figures and Tables

**Table 1:** IC assignment of 78 equine *A. baumannii* isolates with regard to their clinical/organ origin.

Clinical Origin	IC1	IC2	IC3	Non-IC1-IC3
Abscess	0	1	0	1
Eye	1	1	1	0
Feces	1	2	0	0
Gastrointestinal tract	0	1	0	1
Genital tract	0	1	1	18
Organs	0	1	0	3
Respiratory tract	1	1	1	11
Skin, hair, hoof	0	0	0	6
Urinary tract	0	0	1	0
Wound	5	6	1	5
Others	0	2	0	4
Total number of isolates	8	16	5	49

**Table 2:** Antimicrobial resistance phenotype and AMR genes of 78 equine *A. baumannii* isolates.

	Phenotypic Resistance		Genotypic Resistance	
Antibiotic Class	Antibiotic	R (%)	AMR Genes	Positive Isolates (%)
β-lactams	* Ampicillin * Amoxicillin–Clavulanate * Cefalexin Ceftiofur Cefpodoxime Imipenem	100 100 100 98.7 94.9 0	*bla*_ADC-25_ *bla*_OXA-51-like_ *bla*_TEM-1D_	98.7 100 30.8
Aminoglycosides	Gentamicin Amikacin	38.5 1.3	*aadA1* *aa(3)-Ia* *aph(3’)-VIa* *aph(3′)-Ia* *aph(3‘)-Ic* *aph(6)-Id*	29.5 32.1 5.1 23.1 30.8 23.1
Phenicols	* Chloramphenicol	100	*catA1* *ABUW_0982*	26.9 100
Sulfonamides	Trimethoprim–sulfamethoxazole	37.2	*sul1* *sul2*	35.9 2.6
Tetracyclines	Tetracycline	33.3	*tet(A)* *tet(B)* *tet(39)*	10.3 1.3 1.3
Fluoroquinolones	Enrofloxacin Marbofloxacin	37.2 35.9	none ^#^	─
			none ^#^	─
Nitrofuran derivates	Nitrofurantoin	100	none	─

* Intrinsic resistance according to CLSI [25]; ^#^ ENR/MAR-resistant isolates that revealed mutations in GyrA (S83L) (96.7%) and/or ParC (S80L) (92.9%). In the absence of veterinary-specific clinical breakpoints for *Acinetobacter* spp. isolates, human-specific clinical breakpoints from CLSI document M100 [25] were applied whenever possible. For AMC, CPD, NIT, and CHL, breakpoints for *Enterobacterales* [25] were used; for ENR and MAR we used breakpoints provided for ciprofloxacin [25]. For AMP and CEX, breakpoints for *Enterobacterales* as listed by EUCAST [26] were applied. Detailed data about the distribution of phenotypic resistance and AMR genes are provided in Supplementary Table S1.

**Table 3:** Distribution of SBF values of 78 equine *A. baumannii* isolates in LB and M63.

Culture Medium	Incubation Time (h)	SBF		
		Min	Max	Median
LB	24	0.09	25.49	6.61
	48	0.64	20.27	6.01
M63	24	1.75	37.28	12.15
	48	1.64	43.76	12.92

SBF = specific biofilm formation.

**Table 4:** Biofilm-associated genes (BAGs) detected in 78 equine *A. baumannii* isolates.

BAGs	Position in Reference Genome	Product	Function	Positive Isolates (%)				
				All (n = 78)	IC1 (n = 8)	IC2 (n = 16)	IC3 (n = 5)	Non-IC1-IC3 (n = 49)
*abaI*	1396054-1396605	N-acyl-L-homoserine lactone synthetase	Quorum-sensing system: regulates biofilm formation and surface motility	93.59	100	100	100	93.59
*abaR*	1394083-1394799	DNA-binding HTH-domain-containing protein		98.72	100	100	100	98.72
*bfmR*	2304914-2305630	Biofilm-controlling response regulator	Quorum sensing regulated two-component system involved in biofilm formation	100	100	100	100	100
*bfmS*	2305663-2307312	Signal transduction histidine kinase		98.72	100	100	80.00	100
*bap*	536313-541547	Biofilm-associated protein (Bap)	Biofilm formation	55.13	0	100	0	24.36
*blp1*	869025-879134	Bap-like protein 1		0	0	0	0	0
*blp2*	1099370-1101556	Bap-like protein 2		75.64	100	100	100	78.72
*csuA*	3998748-3999194	Csu pilus subunit A	Biofilm formation	93.59	100	100	100	93.59
*csuA/B*	3999371-3999907	Csu pilus major pilin subunit CsuA/B		97.44	100	100	100	97.44
*csuB*	3998224-3998742	Csu pilus subunit B		92.31	100	100	60.00	92.31
*csuC*	3997397-3998230	Csu pilus chaperone protein		98.72	100	100	100	98.72
*csuD*	3994902-3997400	Csu pilus usher protein		98.72	100	100	100	98.72
*csuE*	3993886-3994905	Csu pilus tip adhesin		98.72	100	100	100	98.72
*ompA*	703280-704350	Outer membrane protein A	Antibiotic and serum resistance, biofilm formation, host interaction, cytotoxicity, interference with autophagy and apoptosis	55.13	0	100	0	57.69
*pgaA*	3941315-3942688	poly-beta-1,6 N-acetyl-D-glucosamine export porin	Biofilm formation	100	100	100	100	100
*pgaB*	3938457-3940286	poly-beta-1,6-N-acetyl-D-glucosamine N-deacetylase		98.72	100	100	100	98.72
*pgaC*	3937210-3938457	poly-beta-1,6 N-acetyl-D-glucosamine synthase		97.44	100	100	100	97.44
*pgaD*	3936749-3937213	poly-beta-1,6-N-acetyl-D-glucosamine biosynthesis protein		97.44	100	100	100	93.59

BAGs were identified by screening WGS data of equine *A. baumannii* isolates against BAG sequences of *A. baumannii* reference strains ATCC 17978 (GenBank no. CP018664.1) and AYE (only for *blp1* and *blp2*; GenBank no. CU459141.1).

**Table 5:** Distribution of antimicrobial resistance and AMR genes with regard to biofilm production classification in LB and M73 medium.

Medium	Classification	No. of Isolates	Acquired Phenotypic Resistance to CTU, GEN, ENR, TET, and SXT (0 to 5 Antibiotics) *					
			0	1	2	3	4	5
LB 24 h	non-biofilm	29	0%	44.8%	0%	3.4%	10.3%	41.4%
	weak	43	0%	72.1%	0%	0%	4.7%	23.3%
	moderate	4	0%	25.0%	0%	0%	75.0%	0%
	strong	2	50.0%	50.0%	0%	0%	0%	0%
LB 48 h	non-biofilm	47	0%	46.8%	0%	2.1%	8.5%	42.6%
	weak	26	0%	80.8%	0%	0%	11.5%	7.7%
	moderate	5	20.0%	60.0%	0%	0%	20.0%	0%
	strong	0	-	-	-	-	-	
M63 24 h	non-biofilm	41	2.4%	34.1%	0%	2.4%	4.9%	46.3%
	weak	27	0%	81.5%	0%	0%	7.4%	11.1%
	moderate	10	0%	100%	0%	0%	0%	0%
	strong	0	-	-	-	-	-	
M63 48 h	non-biofilm	44	2.3%	38.6%	0%	2.3%	13.6%	43.2%
	weak	30	0%	83.3%	0%	0%	6.7%	10.0%
	moderate	4	0%	100%	0%	0%	0%	0%
	strong	0	-	-	-	-	-	

* IPM and AMK are not included in the table as all isolates were susceptible.

## Data Availability

Data supporting reported results can be found in the supplementary material. Raw sequence reads of 78 *A. baumannii* genomes are provided under NCBI Bioproject ID PRJNA905332. Further raw data can be made available on reasonable request.

## Supplementary Materials

The following supporting information can be downloaded at: https://www.mdpi.com/article/10.3390/microorganisms11030556/s1, Figure S1: Phylogenetic tree based on the comparison of 2390 core-genome genes of 78 *A. baumannii* isolates from horses and 18 representative *A. baumannii* genomes belonging to international clones IC1-IC9. Figure S2: Alignment of the eight novel OXA-51 variants OXA-970 to OXA-977 in comparison to OXA-51. Table S1: Antimicrobial resistance genes, antimicrobial susceptibility, biofilm-associated genes, and specific biofilm formation of 78 *A. baumannii* isolates from horses. Table S2: Distribution of biofilm-associated genes among equine *A. baumannii* isolates according to their assignment to international clones IC1-IC3 and ST1-ST3.
