# Supplementary material for: Genomic Diversity, Antimicrobial Susceptibility, and Biofilm Formation of Clinical Acinetobacter baumannii Isolates from Horses
Source: Microorganisms. 2023 Feb 22;11(3):556. doi: 10.3390/microorganisms11030556 (PMC10051319; doi:10.3390/microorganisms11030556)
Supplement: Supplementary file 1 [file microorganisms-11-00556-s001.zip › Suppl. Fig. S1_rev.docx]

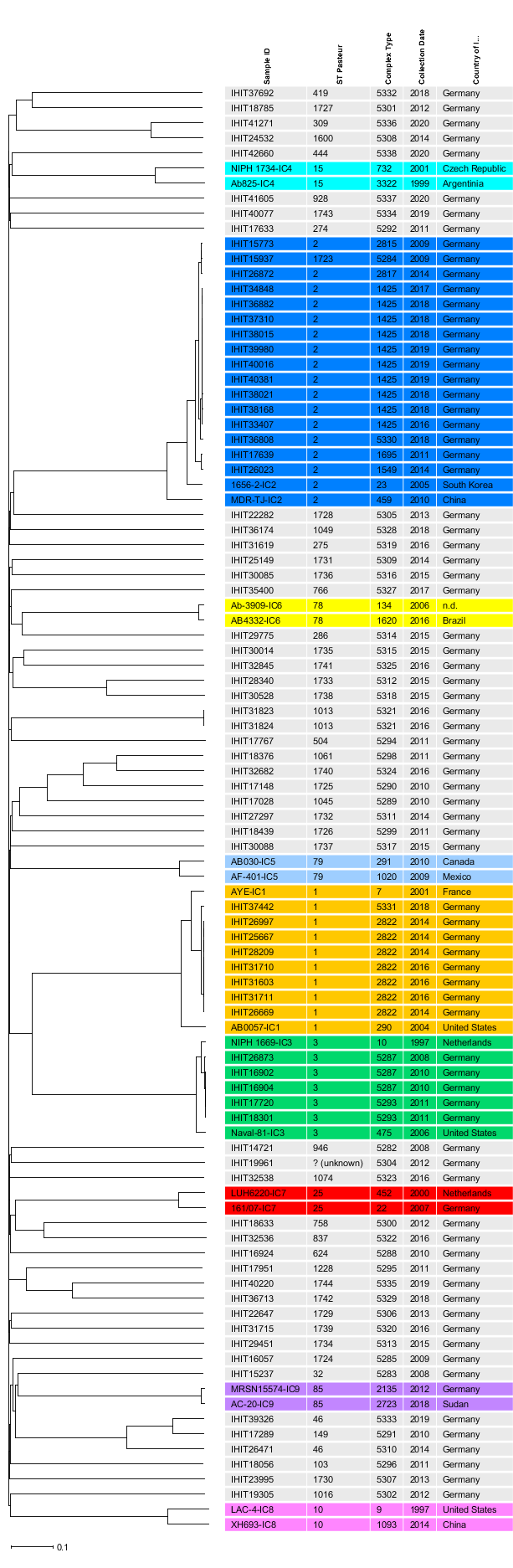
**Supplementary Figure S1:**

Neighbor joining tree (created with Ridom SeqSphere+ v. 7.7.0) based on the comparison of 2390 core genome genes of 78 *A. baumannii* isolates from horses and 18 representative *A. baumannii* genomes belonging to international clones IC1-IC9.

IC4

Groups of clustered isolates are shaded in different colors. Isolates that did not cluster with at least on other isolate or with one of the IC reference genomes are grey-shaded.

Multilocus Sequence Types (ST^Pasteur^), complex types, cgMLST cluster types, sample collection date and country of origin are indicated next to the isolate number.

IC2

IC8

IC9

IC7

IC3

IC1

IC5

IC6
