# Supplementary material for: Genomic Diversity, Antimicrobial Susceptibility, and Biofilm Formation of Clinical Acinetobacter baumannii Isolates from Horses
Source: Microorganisms. 2023 Feb 22;11(3):556. doi: 10.3390/microorganisms11030556 (PMC10051319; doi:10.3390/microorganisms11030556)
Supplement: Supplementary file 1 [file microorganisms-11-00556-s001.zip › Suppl. Fig. S2_rev.docx]

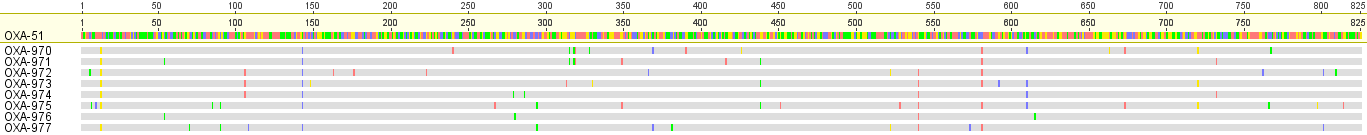


**Supplementary Figure S2:** Alignment based on the amino acid sequences of eight novel OXA-51 variants (OXA-970 to OXA-977; Accession nos. QWA20169.1 – QWA20176.1) from equine *A. baumannii* isolates and OXA-51 reference allele (GenBank: CAC83905.2).
