# Supplementary material for: Genomic Diversity, Antimicrobial Susceptibility, and Biofilm Formation of Clinical Acinetobacter baumannii Isolates from Horses
Source: Microorganisms. 2023 Feb 22;11(3):556. doi: 10.3390/microorganisms11030556 (PMC10051319; doi:10.3390/microorganisms11030556)
Supplement: Supplementary file 1 [file microorganisms-11-00556-s001.zip › Suppl. Tab. S2_rev.docx]

**Supplementary Table S2:** Distribution of biofilm-associated genes (BAGs) among equine *A. baumannii* isolates according to their assignment to international clones IC1-IC3 and ST1-ST3.

| **IC/ST** | **No. of**  **isolates** | **Biofilm-associated genes** | | | | | | | | | | | | | | |
| --- | --- | --- | --- | --- | --- | --- | --- | --- | --- | --- | --- | --- | --- | --- | --- | --- |
|  |  | ***abaI*** | ***abaR*** | ***bap*** | ***blp1*** | ***blp2*** | ***bfmR*** | ***bfmS*** | ***csuA*** | ***csuAB*** | ***csuB*** | ***csuC*** | ***csuD*** | ***csuE*** | ***ompA*** | ***pgaABCD*** |
| **IC1/ST1** | 8 | + | + | - | - | + | + | + | + | + | + | + | + | + | - | + |
| **IC2/ST2** | 15 | + | + | + | - | + | + | + | + | + | + | + | + | + | + | + |
| **IC3/ST3** | 5 | + | + | - | - | + | + | +* | + | + | +** | + | + | + | - | + |

*One IC3/ST3 isolate was negative for the *bfmS* gene; **two IC3/ST3 isolates were negative for the *csuB* gene. BAGs were identified by screening WGS data of equine *A. baumannii* isolates against BAG sequences of reference strain ATCC 17978 (GenBank no. CP018664.1).
